# CHALLENGES AND OPPORTUNITIES FOR TRAINING AND SUPPORTING AIDES AS MEMBERS OF HOME-BASED CARE TEAMS

**DOI:** 10.1093/geroni/igz038.793

**Published:** 2019-11-08

**Authors:** Robyn Stone, Natasha Bryant

**Affiliations:** LeadingAge, Washington, District of Columbia, United States

## Abstract

Despite home heath/home care aides being the informal “eyes and ears” of the health system, team-based home care initiatives have not incorporated this workforce into their programs. This presentation summarizes barriers to their inclusion: a basic lack of understanding on the part of clinical team members of the complex tasks these caregivers perform, inadequate investments in competency-based aide training and education, and variation in state nurse delegation laws that limit aides’ scope of practice and their ability to work effectively in teams. This is followed by a review of several programs that have successfully included aides as key members of home care teams. The presentation concludes with recommendations on how federal and state policymakers, educators and health systems and providers can support inclusion of aides in team-based care through standardization of competency-based training programs, expansion of nurse delegation nationwide, and support for piloting, evaluation, dissemination and replication of promising models.

